# Fire Retardancy of Cementitious Panels with Larch and Spruce Bark as Bio-Admixtures

**DOI:** 10.3390/polym14071469

**Published:** 2022-04-04

**Authors:** Thomas Pacher, Marius Cătălin Barbu, Johannes Urstöger, Alexander Petutschnigg, Eugenia Mariana Tudor

**Affiliations:** 1 Forest Products Technology and Timber Construction Department, Salzburg University of Applied Sciences, Markt 136 a, 5431 Kuchl, Austria; tpacher.htw-m2021@fh-salzburg.ac.at (T.P.); cmbarbu@unitbv.ro (M.C.B.); jurstoeger.htw-m2021@fh-salzburg.ac.at (J.U.); alexander.petutschnigg@fh-salzburg.ac.at (A.P.); 2 Faculty of Furniture Design and Wood Engineering, Transylvania University of Brasov, B-dul. Eroilor nr. 29, 500036 Brasov, Romania; 3 Institute of Wood Technology and Renewable Materials, University of Natural Resources and Life Sciences (BOKU), Konrad Lorenz-Straße 24, 3340 Tulln, Austria

**Keywords:** tree bark, spruce, larch, fire resistance, cement-bonded composite materials

## Abstract

The aim of this study is to investigate the production of fire-resistant panels made out of bark from spruce (*Picea abies*), larch (*Larix decidua* Mill.) and cement. This research included test panels produced from bark, cement, water and cement-bonded recycling material aiming for the target density of 750 kg/m^3^. The physical (density, dimension stability, thickness swelling) and mechanical properties such as tensile strength and compressive strength together with fire resistance were tested. Considering the results, appealing values have been achieved: max. compressive strength: 3.42 N/mm^2^; max. thickness swelling: 5.48%; and density: 515 to 791 kg/m^3^. In principle, the properties of the produced panels depend not only on the density, but also on the hydration and, above all, on the compaction and the composition of the boards. The fire tests demonstrated that the produced panels have an enormous potential in terms of fire resistance and could be utilized for fire-retardant applications.

## 1. Introduction 

Homebuilding, do-it-yourself projects and the manufacturing of home furnishings were the biggest drivers of timber demand worldwide and, as a consequence, the proliferated prices of wooden building materials since 2020. The request on the wood market products at the beginning of 2022 is at a similar rate as in the second quartal of 2021 on the European market [1]. The increasing demands on forestry resources for engineered wood products result in the sparsity of wood supply [2]. The use or reuse of wastes from construction and wood industry sector, together with sustainable technologies, are included in the bio-economy strategy the European Union through a bio-based and circular economy [3]. Alternative lignocellulosic raw materials and an improved utilization rate of wood resources are key elements of this road map [4]. There is a large potential to use biomass and minerals in a composite matrix with fire-retardant properties [5]. Current nonflammable products on the construction market, mostly with inorganic material, have a high performance, but the environmental impacts in their production processes are significant [6]. 

Beside its hydrophilic nature [7,8], another disadvantage of lignocellulosic material is low fire resistance due to (C, H, and O) in the cellulose structures that boost to initiate combustion [9]. Flame-retardant additives have been extensively investigated for construction materials [10,11] as substances that impede the fire propagation in a material [12]. The adjoining of clay [13] and cement [14,15] in lignocellulosic composites can contribute to the fireproof properties due to the effects of reducing heat transfer and catalyzing the char development from cellulose due to the metal ions included in the chemical composition of these materials [5]. Cement bound wood-wool panels have been on the construction market since the beginning of 20th century [16]. Wood-wool can be replaced successfully with tree bark in such applications [13]. The phenolic compounds in tree bark also provide fire-retardant properties to such composites, together with the protective role of bark as fire-stopping found in many species [17]. As a by-product of wood processing industry, tree bark has an enormous potential in applications as adhesive filler to reduce formaldehyde emissions [18], thermal [19,20] and acoustic insulations [21], as raw materials for MDF [22], particleboard [23], pellets [24] or for medicinal purposes [25]. The combination between tree bark and cement results in lightweight concrete composites with densities around 800 kg/m^3^ [14]. Wood and tree bark, due to hemicelluloses, starches, sugars, phenols and acids, tend to hinder cement hydration in composite, resulting in longer setting time and a limitation in the strength of material, as a consequence of micro-fracturing of the matrix during cement hydration [26,27]. There are several studies that dealt with bark-cement composites. Thermal insulation properties of cement-based admixtures with tree bark were analyzed by [28,29], in cork composites with slag cement. Other research involving cement bound cork granules for the manufacture of lightweight composites was carried out by [30]. The influence of *Eucalyptus globulus* bark fiber on the mechanical properties of cementitious composites were studied by [31]. It was stated that such composites can find application in numerous fields, from agriculture to roadways and tunneling. The inhibitory role of the bark on the cement’s hydration was assessed with sugi bark (*Cryptomeria japonica*) by [32]. It was found that the addition of magnesium chloride and sodium silicate significantly improved the hydration behavior of materials and, in some cases, of the compressive strength. Urstöger et al. [14] examined the compressive strength and hydration behavior of composites with spruce and larch bark bounded with cement for lightweight composites and emphasized the larch bark influence on composite properties, especially in cold pressing system. This research is a follow-up of [14] and deals with the flammability analysis of cement bound larch and spruce bark composite panels, besides recycled material from previous experimental trials.

## 2. Materials and Methods

The raw materials used in this study were spruce (S) (*Picea abies*) and larch (L) (*Larix decidua* Mill.) bark, sourced from Rupert Deisl Co. sawmill in Adnet, Austria. The bark material was shredded with a R40 four-spindle shredder (Untha shredding technology Co., Kuchl, Austria), dried to a moisture content (MC) of 14–15% in an exhaust air drying system (Hildebrand-Brunner, Hannover, Germany) and then fractionated into particles < 3 mm, 3–7 mm and 7–12 mm. The binder applied was Portland-composite cement CEMIIA-LL42.5N ‘PROFI-CEM’ from Zementwerk Leube Co. (St. Leonhard, Austria) and recycled material from cement-bonded bark with a particle size 1 < x < 4 mm, which originated from preliminary trials. These trials served as prerequisites for this study, to find out the appropriate composition of the panels (bark, water and cement amount) at a target density of 750 kg/m^3^. The cement-bark mixture from preliminary trials was reused in this study, at a constant percent of 6.4% for all the boards. This issue emphasized the role of recycling material in the manufacturing process of cementitious bark-based panels, combining here two elements of circular economy concept: reuse (bark/cement mixture) and upcycling of a secondary product of wood industry, namely tree bark.

### 2.1. Sample Preparation

**Figure 1:**
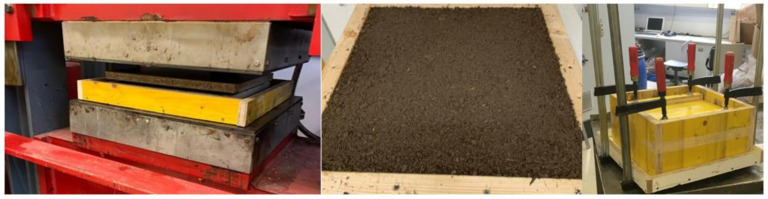
Board compaction by hydraulic press (**left**); test specimens 430 × 430 × 20 mm in a forming frame (**middle**); and board compaction by screw clamps (**right**).

In order not to influence the water-binder value (w/b value), the moisture content (MC) of the bark types was raised at the beginning of the experimental trials from 14–15% to approximately 100% with a 24 h water storage. Subsequently, the bark particles were mixed with cement and recycled material in a single-shaft ploughshare mixer ENT type WHB-75 (WAMGROUP S.p.A., Ponte Motta, Italy). The water was added after the “dry” components formed a homogeneous mass. Four test groups (TG), depending on the particle size and the compaction method were used; recycled material was added to all TGs, this resulted from preliminary trials and was added in a proportion of 6.64% in all panel formulations. The compaction was realized with conventional screw clamps (SC) and a hydraulic press Höfer HLOP 280 (Höfer, Taiskirchen, Austria) (HP) at room temperature (RT, 20 °C) (Table 1 and Figure 1).

Depending on the compaction method (SC or HP), the cement bound bio-aggregates were closed in molds for one week with SC or pressed (at 20 °C) for 6 to 8 min with the Höfer HLOP 280 hydraulic laboratory press with a specific pressure between 0.75 and 2.0 N/mm^2^ (Table 1). For curing, all samples were stored in a standard climate at 20 °C and 65% relative humidity for 28 days.

### 2.2. Fire-Resistance Test

**Figure 2:**
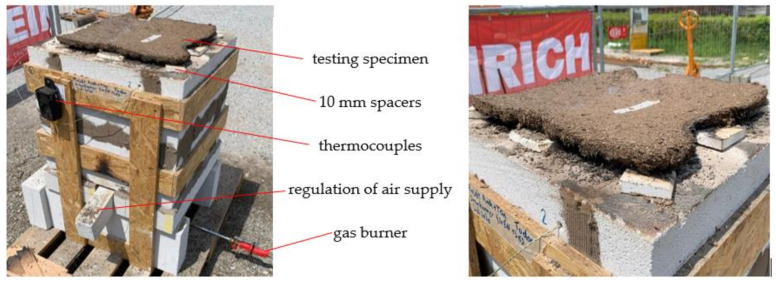
Set-up of the experimental design of the fire resistance measurement with the help of the furnace (**left**); and test object during the performance of the experiment on the furnace (**right**).

The furnace was built according to fire resistance test EN 1363-1:2020 [33] by [34,35] with porous concrete blocks. The cement-bonded bark panels were set on the top of the furnace (Figure 2 left). To ensure a sufficient ventilation, spacers of 10 mm were used between the panel and the furnace. At the right bottom side of the furnace, a gas burner was placed, which indirectly flamed the material from below. The temperature was measured with four thermocouples (two in the upper and two in the lower part of the combustion chamber) connected to a data logger device. At the beginning of the test, the surface temperature of the specimen was determined and then measured every five minutes. The classification into fire classes is based on the time needed from test object to reach the surface temperature by 140 °C (thermal insulation), starting from the initial temperature. For the determination of the integrity, the time needed to reach a temperature increase of 260 °C is essential, and finally the time until the surface of the cementitious composite reaches 300 °C was also measured (Figure 2 right).

## 3. Results

After 28 days, the composite boards were completely cured. Afterwards, the panels were cut to size (EN 326-1:2005 [36]) to determine the physical (moisture content, thickness swelling and density) and mechanical properties (compressive and tensile strength). In order to represent the scatter within the panel and to obtain reliable test results, the test specimens were cut from different areas of the panel.

### 3.1. Determination of the Physical Properties

The physical properties tested for the bark-cement panels were moisture content, thickness swelling and density (Table 2). The MC was determined according to EN 322:2005 [37], the thickness swelling to EN 317:2005 [38] and the density to EN 323:2005 [39].

#### 3.1.1. Moisture Content (MC)

The MC of panels made out of larch bark is generally higher in comparison to those of spruce bark. The test specimens of spruce bark achieved a total average MC of 13.15%, compared to 15.17% for larch bark (Table 2) due to the differences on chemical composition: 28.8% lignin and 36% polysaccharides for larch bark [40] and 26.9% lignin and 47.9% polysaccharides [41] for spruce bark.

#### 3.1.2. Thickness Swelling (TS) after 24 h Water Soaking

**Figure 3:**
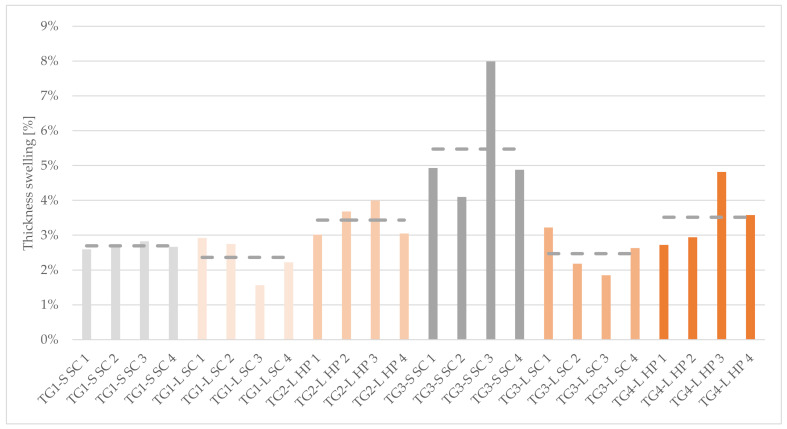
Thickness swelling after 24 h water soaking of 50 × 50 × 20 mm cement-bonded bark specimens. Note: TG, test group; S, spruce; L, larch; SC, screw clamps; HP, hydraulic press.

The larch bark panels achieved an average TS of 2.95%. TG1-S SC reached an average of 2.69%, while samples of TG3-S SC recorded a mean of 5.48%. Another aspect of TG3-S SC is an outlier to 8% (TG-S SC 3) while the rest of the samples swelled in thickness with 4.5%. Conspicuous is that the compaction method (SC or HP) influenced this property. As the results from Table 2 and Figure 3 show, the values for TG2-L HP and TG4-L HP are 1% higher than those with SC (when using larch bark).

Another characteristic of this test is the different behavior of the cement-bonded test specimens made out of spruce bark compared to the larch bark samples. While measurement of TS was possible for the specimens TG1-S SC and TG3-S SC, the series TG2-S HP broke during the cutting and TG4-S HP during the water storage. For this reason, it was not possible to carry out the tests. The samples of TG3-S SC yielded values almost twice as high as those of TG1-S SC, which can be attributed to the different bark-fractions, nevertheless this finding cannot be stated for other TGs. The standard deviation (SD) in TG3-S SC is also significantly higher. This suggests a widely spread of the TS values of this TG. The differences of the compaction methods are illustrated in Figure 3. Samples of TG1-L SC achieved lower TS than those of TG2-L HP. This finding can also be confirmed in the comparison between TG3-L SC and TG4-L HP.

#### 3.1.3. Density

**Figure 4:**
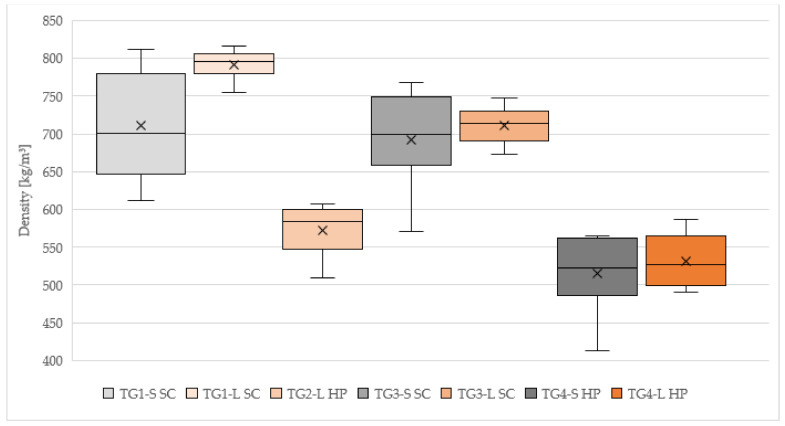
Density of 50 × 50 × 20 mm cement-bonded bark specimens. Note: TG, test group; S, spruce; L, larch; SC, screw clamps; HP, hydraulic press.

The results of the average density measurements are shown in Table 2 and Figure 4. The samples of TG1-S SC reached on average 710 kg/m^3^, with the highest SD of all test groups. Test specimens of TG3-S SC measured a density of 692 kg/m^3^, TG4-S HP had the lowest density of all TGs with 515 kg/m^3^. In case of larch bark, the highest values were achieved with samples from TG1-L SC (791 kg/m^3^). For TG2-L HP the densities had an average of 572 kg/m^3^. TG3-L SC (711 kg/m^3^) obtained almost identical results as TG3-S SC (692 kg/m^3^), as well as TG4-L HP (530 kg/m^3^) and TG4-S HP (515 kg/m^3^).

The densities of the test specimens compressed with HP were far lesser compared to the press system with SC. In both test groups (TG2 and TG4), significantly lower values as for samples compressed with SC (TG1 and TG3) were obtained. Compared to the target density of 750 kg/m^3^, almost all values are lower, as a consequence of evaporation of water from cement during the curing process.

Density is one of the most important factors influencing the mechanical properties of the specimens, higher values leading to an increase in compressive strength [42] The density of the cement bonded panels with bio-aggregates ranged between 515 and 791 kg/m^3^. For further investigations, the density is an important influencing parameter for the mechanical behavior and could be increased for new testing groups, as it is usual for cement-bonded wood-based materials (800 kg/m^3^).

### 3.2. Determination of the Mechanical Properties

**Figure 5:**
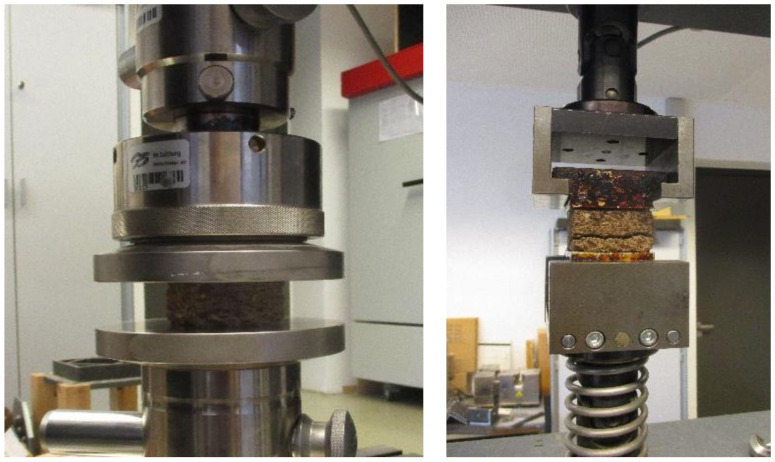
Compressive strength measurement at 10% compression of the 50 × 50 × 20 mm cement-bonded bark specimens using universal testing machine Zwick/Roell 250 (**left**); internal bond measurement of the 50 × 50 × 20 mm cement-bonded bark specimen using universal testing machine Zwick/Roell 250 (**right**).

The mechanical properties analyzed in this research are compressive strength and transverse tensile strength. The compressive strength (the most relevant test for cementitious composites) was determined according to EN 826:2013 [43] with samples of 50 mm × 50 mm × 20 mm and the transverse tensile strength (internal bond, that express the stability of the composite panels and the quality of the bonding between bark particles and cement) to EN 319:2005 [44] with 50 mm × 50 mm × 20 mm samples fixed with hot melt glue and disposed at 90° in the testing clamps. The mechanical properties of the test specimens were measured with the universal testing machine Zwick/Roell 250 (Ulm, Germany) (Figure 5).

#### 3.2.1. Compressive Strength

The highest average compressive strength (Figure 5 left) at 10% compression was achieved in TG1-L SC with 3.17 N/mm^2^ (Table 3). For TG3-L SC, the mean value was 2.87 N/mm^2^, this TG showed the highest compressive strength of all test specimens with 3.43 N/mm^2^. The samples of TG2-L HP had a compressive strength of 0.22 N/mm^2^, TG4-L HP measured 0.32 N/mm^2^ and TG4-S HP had a compressive strength of 0.27 N/mm^2^.

**Figure 6:**
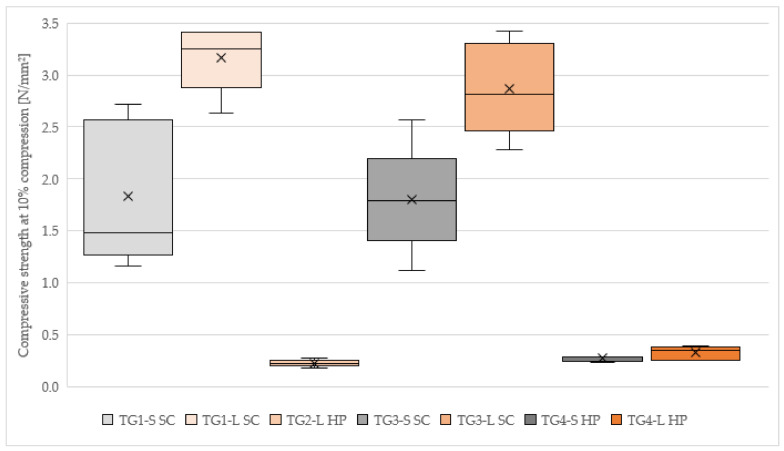
Compressive strength at 10% compression of 50 × 50 × 20 mm cement-bonded bark specimens. Note: TG, test group; S, spruce; L, larch; SC, screw clamps; HP, hydraulic press.

As results from Figure 6, there are differences between the TGs: for the panels compressed with SC the compressive strength ranged from 1.12 to 3.43 N/mm^2^. The hydraulic-pressed panels did not achieve any values higher than 0.39 N/mm^2^. The samples from TG1-L SC and TG3-L SC achieved better results, which is only partially the case for TG2-L HP and TG3-L HP. Based on these results, no tangible conclusion can be drawn about a different behavior regarding the influence of bark particle size on the compressive strength.

**Figure 7:**
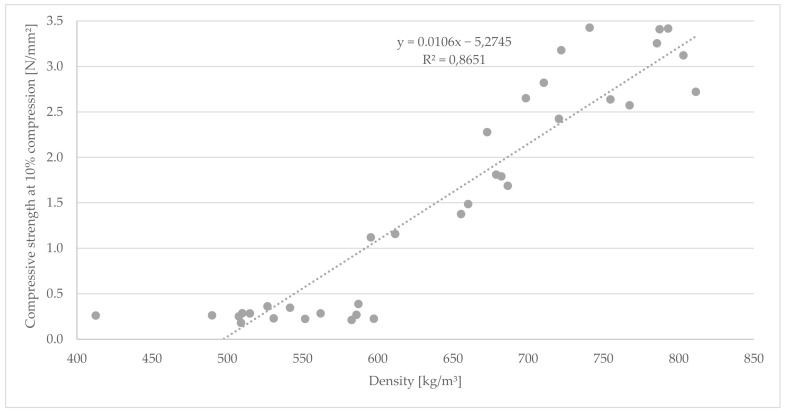
Correlation between compressive strength at 10% compression and density for the cement bonded bark panels.

As shown in Figure 7, there is a significant positive correlation (R^2^ = 0.87) between density and compressive strength for all TGs. This is in accordance with the general fact that density has a considerable influence on the mechanical properties of composite materials [42].

#### 3.2.2. Internal Bond (IB)

**Figure 8:**
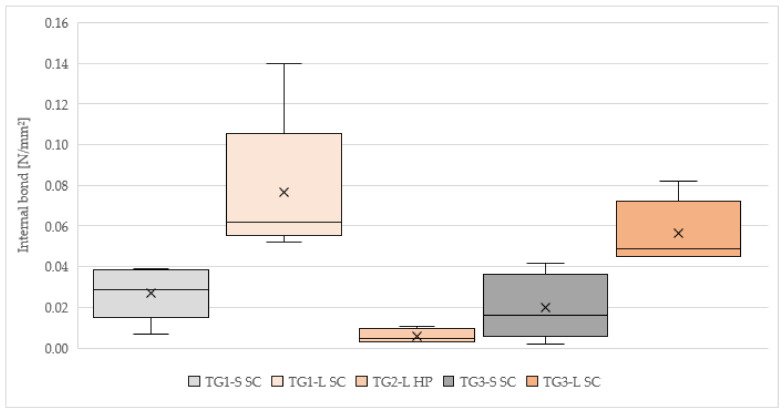
Internal bond of 50 × 50 × 20 mm cement-bonded bark specimens. Note: TG, test group; S, spruce; L, larch; SC, screw clamps; HP, hydraulic press.

The values of IB were less than 0.14 N/mm^2^, which can be attributed to the brittleness and porosity of the panels (Figure 5 center and Figure 8). When comparing the bark species, larch bark composites measured at least two-fold values compared to the cement admixtures with spruce bark aggregates.

The specimens in TG1-L SC achieved an average IB of 0.08 N/mm^2^, those of TG1-S SC only 0.03 N/mm^2^ (σ = 0.01 N/mm^2^). In TG2, only the samples made of larch bark could be tested, while those made of spruce broke/failed during cutting. The specimens of TG2-L HP had the lowest IB, under 0.01 N/mm^2^. In TG3, the results were comparable to those in TG1, whereby both bark types could not reach the strength achieved in TG1-L SC with 0.06 N/mm^2^ and TG1-S SC of 0.02 N/mm^2^. No data could be determined for the test specimens TG4-S HP and TG4-L HP due to the increased brittleness of the samples, which failed shortly after the test start.

**Figure 9:**
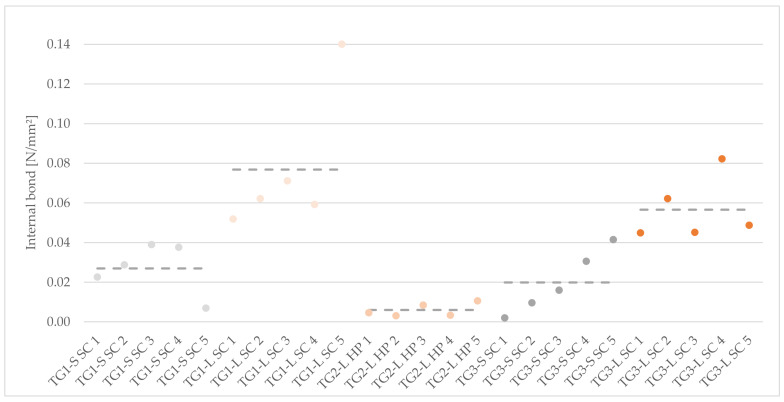
Distribution of internal bond within the TGs of 50 × 50 × 20 mm cement-bonded bark specimens. Note: TG, test group; S, spruce; L, larch; SC, screw clamps; HP, hydraulic press.

The highest results of IB were achieved with TG1-L SC and reached values similar to other mineral-bonded bark materials [13]. Samples of TG1-S SC (fraction < 3–12 mm) obtained higher results compared to TG3-S SC (fraction < 3–7 mm). There are partially large deviations between the test specimens. For this reason, the individual results are shown in Figure 9, in order to better recognize the scattered values within the test groups. A number of results are clearly above the average. However, the IB of the specimens is generally very low, but represents a characteristic of mineral-bonded bio-aggregates [13,15].

IB is strongly related to particle distribution, bonding and compaction inside the panels [14], but can also be caused by incorrect or incomplete curing of the cement [45]. In case of specimens with spruce bark, the low IB can be attributed to incomplete curing of the cement, in some cases the specimens failed even without the application of force during the test preparation (Figure 5 right). The influence of the different cellulose content (due to biodegradation during storage) in the bark might also play a significant role within the mixture [46].

The test specimens were porous, this resulted in an inhomogeneous bond between the bark fractions and the cement [47], which is also reflected in the low IB, especially of samples from TG2-L HP. The pressing method could also have a significant influence, the samples compacted with HP showed lower values compared to the compaction using SC [14]. It is possible that too much pressure during pressing is not necessary and reduces the panel properties.

Failures of the test specimens were already detected after minimal loads, which can be described by various parameters for examples the hydration or the different chemical ingredients [30]. The findings of the experimental trials also showed that higher IB was not obtained by pressing with HP. Too high pressure can have a negative influence on the mechanical properties due to breakage of particles. The hydration of the panel is also essential for this test, because if there is an incorrect curing inside the panel, it is not possible to ensure an appropriate bonding of the bark particles [14]. As a further aspect, the generally low IB of concrete can be mentioned, as described by [48].

#### 3.2.3. Fire Resistance

For the determination of the fire resistance, one test panel with the dimensions of 430 × 430 mm was used per test group. Due to the considerable complications that arose during the removal of the cementitious panels from the molds and their manipulation and preparation of experimental setup, not all test groups could be tested for fire resistance. The test was carried out according to EN 1363-1:2020 [33], so the results give a rough overview on the properties of the panels in terms of fire resistance, but are not comparable with established products tested under standardized conditions [10].

**Figure 10:**
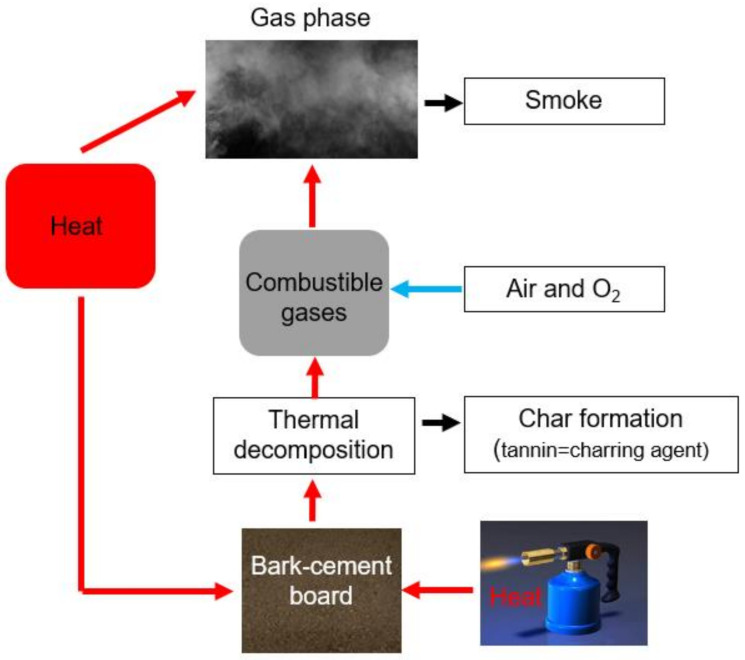
Flame-retardant mechanism of cementitious panels with spruce and larch bark.

The flame-retardant mechanism of the bark cement-boards (Figure 10) is mainly due to mineral enriched matrix in the composites. Cement belongs to the class of non-combustible building materials according to DIN 4102-4:2004 [49]. On the other side, bark is a natural barrier of tree against fire. In regions more prone to fire, resistant and thicker barks that protect the underlying meristematic tissues against high temperatures were developed [17]. The main components of bark that inhibit fire are graphite, inserted and distorted graphite–graphene aggregates [50] and polyphenols (tannins). The tannins contribute to the generation of graphite during charring. Moreover, tannins have high molecular weights (up to 20,000) and form complexes with proteins and alkaloids, that precipitate. The polyphenols form complexes with polysaccharides too and perform as antioxidants. Moreover, tannins are able to neutralize radicals due to their endowment to donate electrons [51]. Through electron donation, tannins determine reduction of heat power, which could retard and slow down the fire.

Due to the different external influences and the fact that the tests took place outdoors, the temperature curve could not be achieved as described in the standard [33]. During this experiment, test panels of TG1-L SC, TG2-L HP, TG3-L SC, TG4-L HP, TG4-S HP and a gypsum reference could be tested with regarding to the fire resistance, including thermal insulation and integrity.

**Figure 11:**
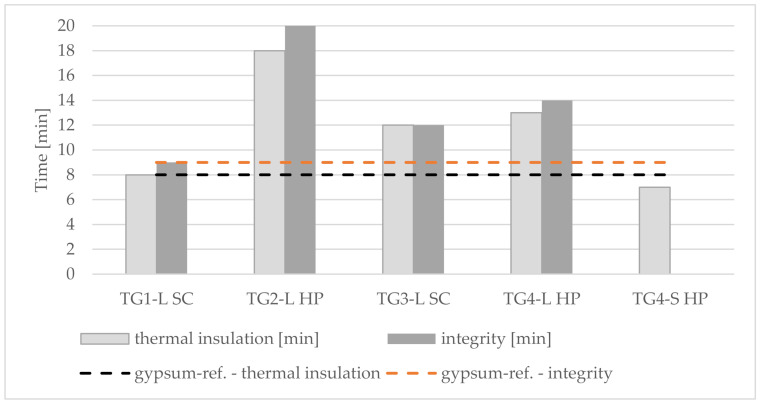
Comparison of the cement bonded bark test specimens with gypsum reference panel (430 × 430 × 20 mm). Note: TG, test group; S, spruce; L, larch; SC, screw clamps; HP, hydraulic press.

The test panel from TG2-L HP achieved the most representative result with a time of 18 min for thermal insulation (étanchéité). Noticeably, the temperature increase was slow at the beginning, but as soon as the first temperature limit (140 °C) was reached, a rapid rise was developed on the backside of the panel. The time difference between reaching the limits for thermal insulation (140 °C), integrity (260 °C) and the end of the test was measured minute by minute. For TG4-S HP (*) (Table 4 and Figure 11) it was not possible to make a reliable measurement, as the panel failed after 7 min at a temperature of 140 °C and began to crack at different spots. Due to complications during hydration, the test specimen of TG3-S SC broke as soon as it was placed on the top of the combustion chamber.

**Figure 12:**
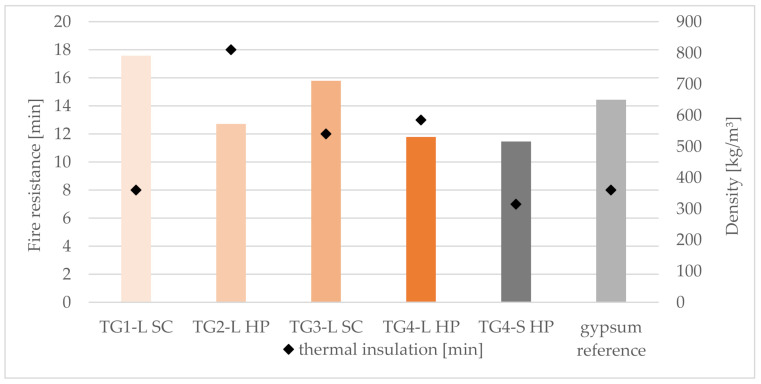
Relationship between density, fire resistance and thermal insulation cement-bonded bark panels. Note: TG, test group; S, spruce; L, larch; SC, screw clamps; HP, hydraulic press.

Due to the partly large deviation from the standard fire curve and the acceptable results regarding fire resistance, it should be admitted that significantly longer test times are possible if the tests were carried out strictly according to the standard (EN 1363-1:2020). In this case, the values of the thermal insulation were used to determine the fire resistance (Table 4 and Figure 12).

**Figure 13:**
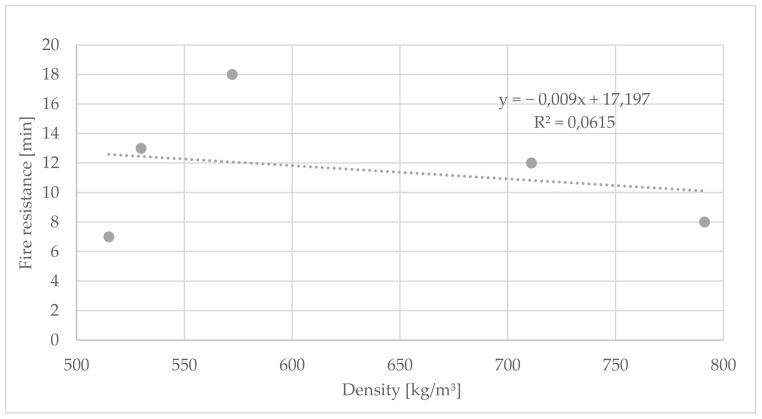
Correlation between fire resistance duration and density of cement-bonded bark panels.

There is no correlation (R^2^ = 0.06) between the density and the fire resistance duration (Figure 13). The assumption is that panels with higher densities have a higher cement content (>40%) and therefore more fire-retardant material is available [15].

The fire resistance measurements delivered acceptable results irrespective of the high deviation from the standard fire curve. External influences during these outdoor tests, i.e., weather conditions (wind), can be mentioned as important influencing parameters. Due to fire protection regulations, the test could not be carried out inside the campus facilities, so it was necessary to select a wind-protected location and select testing days without significant wind for the test setup to limit the other influencing parameters, such as the air current and temperature outside.

The evaluation of the test provided interesting results regarding the correlation between density and fire resistance duration.

**Figure 14:**
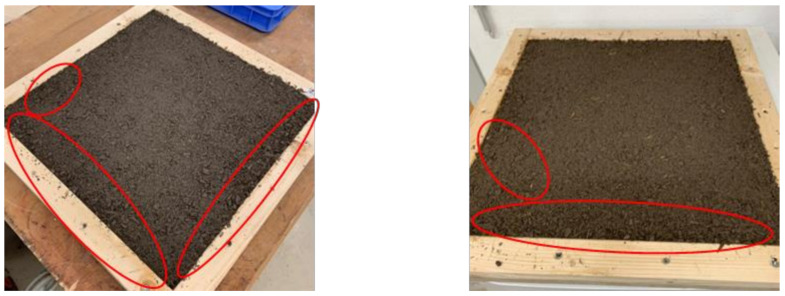
Cement bonded bark test specimens (430 × 430 × 20 mm) in a forming frame, nests with large bark particles marked in red.

The longest thermal insulation (resistance) was achieved with TG2-L HP (18 min.) (Figure 12). The reason for this is on the one hand the fine and compact structure of the cement admixtures with bark aggregates, which protects well against heat, and on the other hand the acceptable curing behavior of the panel. Furthermore, it was observed that in the case of panels with nests of large bark particles (Figure 14), the burn-through occurs rapidly, especially in these areas. The panel heats up from these clusters, so the temperature limits are reached quickly.

## 4. Conclusions

The fire behavior of cementitious composites with spruce and larch bark is strongly influenced by the constituents synergy. Cement as non-flammable construction material (A1 classification) in the matrix was mixed with bio-aggregates made of tree bark, known for its protective role for the tree. The tannin content of softwood bark helps reducing the heating power of the flame, acting as charring agent, by forming a protective layer against the heat and against diffusion to the flame of combustible volatile compounds. The significant variation of tannin amount in the analyzed bark species leads to the conclusion that more studies are required to find out in which way the fire retarding mechanism can be improved by adding other components to the panel´s formulation.

This composite type may provide an option for using lignocellulosic residues with improved properties as fire retardancy with application for example as wall covering and filling material in constructions sector. The use of mineral-bonded bark-based composites is highly dependent on building regulations codes, construction specifications, esthetics, and all the other factors which influence public approach of a product.

The experimental trials with cement-bonded panels with bio-aggregates of spruce and larch bark showed that test groups with larch bark pressed with screw clamps (TG-L SC) had the greatest potential regarding to the performed tests. In the case of applying hydraulic pressing (HP), it resulted that further investigations of the pressing parameters such as temperature, pressure and time are necessary to continuously optimize the quality and strength of the panels. Based on the available values for HP, it can be assumed that the pressing time was too short to ensure sufficient compaction of the panel. Additional investigations should be conducted to determine the influence of the pressing time and, if appropriate, the molds can be weighted after pressing and during curing. The different chemical constituents of the bark types (lignin, holocellulose, extractives) also provide an interesting base for further research and could have different influences on the setting behavior, as noticed between the samples made of spruce (TG-S) and larch (TG-L). The amount of holocellulose in bark negatively influences the compatibility between cement and bio-aggregates.

The quantity ratio (w/b ratio) (Table 1) was kept constant at 0.5, but this aspect still offers enormous potential for further optimization. At the same time, it would be useful to reduce the cement proportion to a minimum, thus saving valuable resources and reducing costs in order to create an economically attractive product. As an alternative, the percentage of bark and recycled material should be consequently observed, which will also result in a higher proportion of sustainable material in the end-product.

The different fractions of the bark (<3–12 mm and <3–7 mm) presented no significant influence with regard to the physical and mechanical properties of the panels. Correct hydration [14] can be considered as another important factor, which is why preliminary tests should be carried out to find other appropriate types of bark. Furthermore, the different pH values of bark can also have a relevant influence on hydration.

## Figures and Tables

**Table 1:** Experimental design for bark-based cement-bonded composites. Note: Comp., Compaction; RM, Recycling Material; Temp., Temperature; SC, screw clamps; HP, hydraulic press; h, hours; min, minutes.

	Bark	Comp.	Particle Size (%)			Cement (%)	Water (%)	RM (%)	Press Parameter		
			<3 mm	3–7 mm	7–12 mm			<1–4 mm	Pressure (N/mm^2^)	Temp. (°C)	Time
TG1 <3–12 mm	spruce	SC	9.86	12.30	12.30	39.26	19.64	6.64	-	20	~168 h
	larch										
TG2 <3–12 mm	spruce	HP	9.86	12.30	12.30	39.26	19.64	6.64	0.8–2.0	20	6–8 min
	larch										
TG3 <3–7 mm	spruce	SC	9.86	24.60	-	39.26	19.64	6.64	-	20	~168 h
	larch										
TG4 <3–7 mm	spruce	HP	9.86	24.60	-	39.26	19.64	6.64	0.75	20	6–8 min
	larch								1.10		

The tests were carried out at a w/b value of 0.5 and a target density of 750 kg/m^3^, with a panel size of 430 × 430 mm and a thickness of 20 mm.

**Table 2:** Moisture content, thickness swelling and density of cement-bonded bark panels with spruce and larch bark and two types of bark particle sizes. Note: TG, test group; SC, screw clamps; HP, hydraulic press; standard deviation in parentheses.

	Bark	Compaction	Moisture Content (%)	Thickness Swelling (%)	Density (kg/m^3^)
TG1 <3–12 mm	spruce	SC	13.06	2.69 (0.10)	710 (74)
	larch		15.89	2.36 (0.61)	791 (20)
TG2 <3–12 mm	spruce	HP	-	-	-
	larch		14.65	3.43 (0.48)	572 (32)
TG3 <3–7 mm	spruce	SC	13.34	5.48 (1.72)	692 (66)
	larch		16.07	2.47 (0.59)	711 (25)
TG4 <3–7 mm	spruce	HP	13.06	-	515 (56)
	larch		14.06	3.52 (0.94)	530 (37)

**Table 3:** Compressive strength and internal bond of cement-bonded bark panels with spruce and larch bark and two types of bark particle sizes. Note: TG, test group; SC, screw clamps; HP, hydraulic press; standard deviation in parentheses.

	Bark	Compaction	Test Samples	Compressive Strength (N/mm^2^)	Internal Bond (N/mm^2^)
TG1 <3–12 mm	spruce	SC	n = 5	1.83 (0.69)	0.03 (0.01)
	larch		n = 5	3.167 (0.320)	0.08 (0.036)
TG2 <3–12 mm	spruce	HP	n = 5	- *	- *
	larch		n = 5	0.221 (0.032)	0.006 (0.003)
TG3 <3–7 mm	spruce	SC	n = 5	1.795 (0.52)	0.020 (0.016)
	larch		n = 5	2.870 (0.45)	0.057 (0.016)
TG4 <3–7 mm	spruce	HP	n = 5	0.268 (0.02)	- *
	larch		n = 5	0.321 (0.06)	- *

* The measurement was not possible due to the failure of the testing specimen (very brittle).

**Table 4:** Results of the fire resistance measurement of the 430 × 430 × 20 mm cement-bonded bark test samples and a gypsum reference panel. Note: TG, test group; S, spruce; L, larch; SC, screw clamps; HP, hydraulic press; *, failed during test.

Fire Resistance According to EN 1363-1:2020	TG1-L SC	TG2-L HP	TG3-L SC	TG4-L HP	TG4-S HP	Gypsum Reference
Furnace temp.—start (combustion chamber ≈ 100 °C)	110.0 °C	109.6 °C	113.5 °C	121.4 °C	121.3 °C	91.4 °C
Surface temp—start	32 °C	34 °C	35 °C	37 °C	34 °C	25 °C
Thermal insulation (surface temp. + 140 °C)	8 min	18 min	12 min	13 min	7 min *	8 min
Integrity (surface temp. = 260 °C)	9 min	20 min	12 min	14 min	-	9 min
End (surface temp. = 300 °C)	10 min	22 min	13 min	15 min	-	10 min

## Data Availability

Not applicable.
